# Supplementary material for: Assessment of Genetic Stability in Human Induced Pluripotent Stem Cell-Derived Cardiomyocytes by Using Droplet Digital PCR
Source: Int J Mol Sci. 2024 Jan 16;25(2):1101. doi: 10.3390/ijms25021101 (PMC10815998; doi:10.3390/ijms25021101)
Supplement: Supplementary file 1 [file ijms-25-01101-s001.zip › ijms-2807415-supplementary.pdf]

Supplementary Files

Table S1. Primer sequences.

Genes	Sequence (5′-3′)	Length (bp)
POU5F1-F	GACAGGGGGAGGGGAGGAGCTAGG	24
POU5F1-R	CTTCCCTCCAACCAGTTGCCCCAAAC	26
ISL1-F	AGATCAGCCTGCTTTTCAGC	20
ISL1-R	AGGACTGGCTAACCATGCTGT	21
TNNT2-F	ATGAGCGGGAGAAGGAGCGGCAGAAC	26
TNNT2-R	TCAATGGCCAGCACCTTCCTCCTCTC	26
MYL2-F	ACAGGGATGGCTTCATTGAC	20
MYL2-R	ATGCGTTGAGAATGGTTTCC	20
MYH7-F	ACATGCTGCTGATCACCAAC	20
MYH7-R	AAGCGTTATCAGTGGCCATG	20
MYL7-F	GGAGTTCAAAGAAGCCTTCAGC	22
MYL7-R	TCCTCTGGGACACTCACCTT	20
MYH6-F	TTCTCCGTGAAGGGATAACC	20
MYH6-R	CGTCTTCCCATTCTCGGTTTCAGC	24
18s rRNA-F	AGGAATTGACGGAAGGGCACCA	22
18s rRNA-R	GTGCAGCCCCGGACATCTAAG	21

[1] F: Forward
[2] R: Reverse

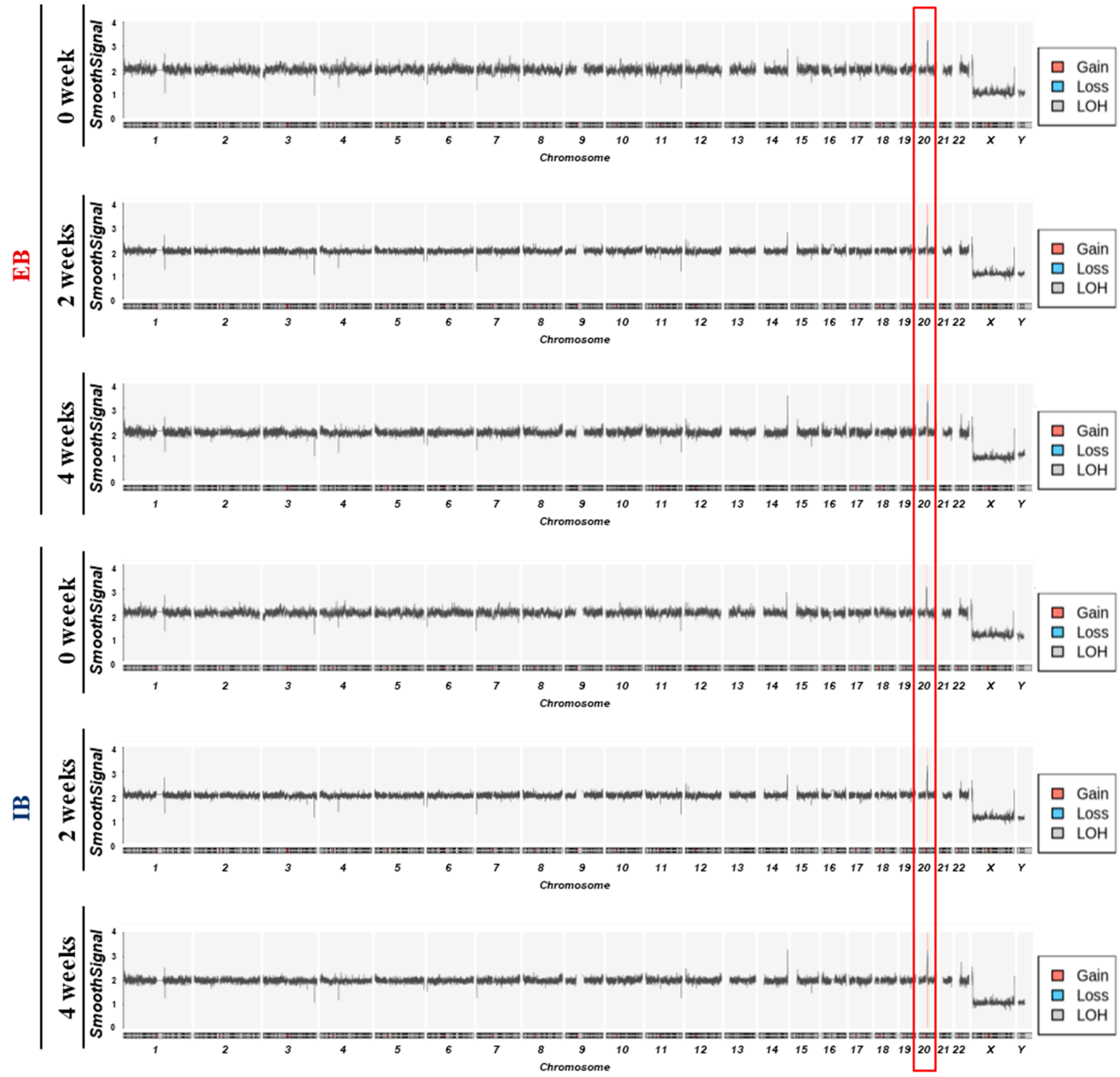

Figure S1. CytoscanHD chip analysis in hiPSC and hiPSC-CM.

(A) KMT2C

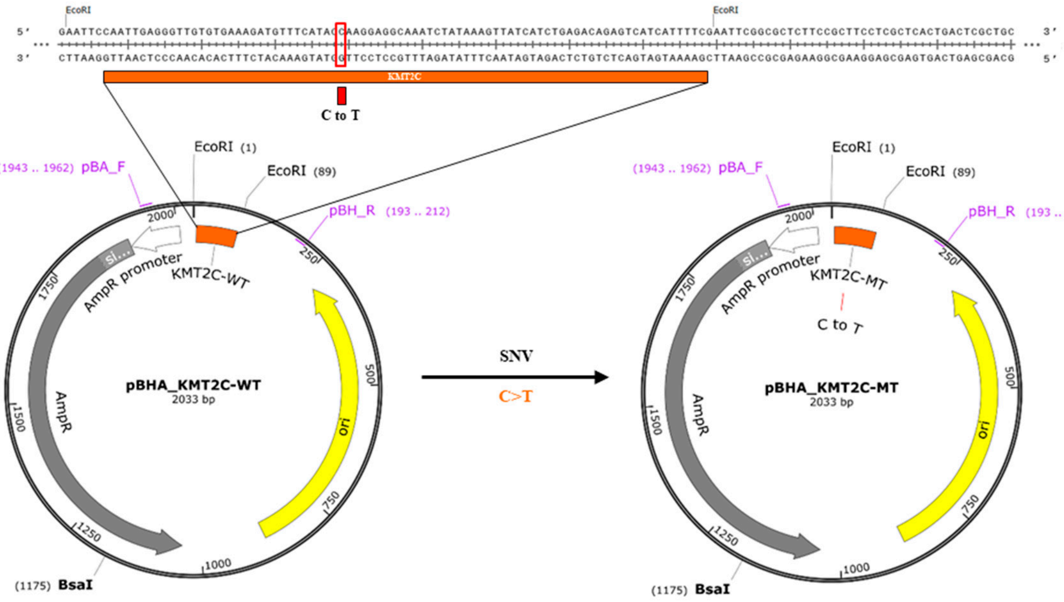

(B) BCOR

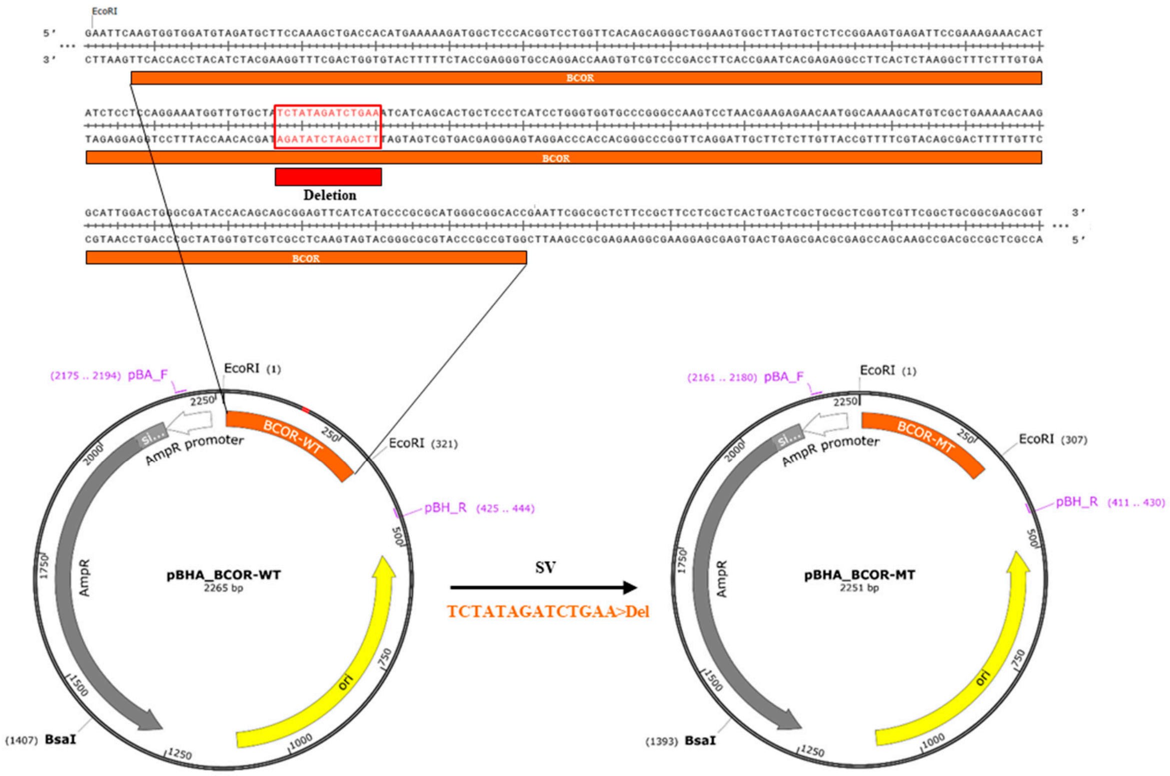

Figure S2. Plasmid vector cloning for control template design.

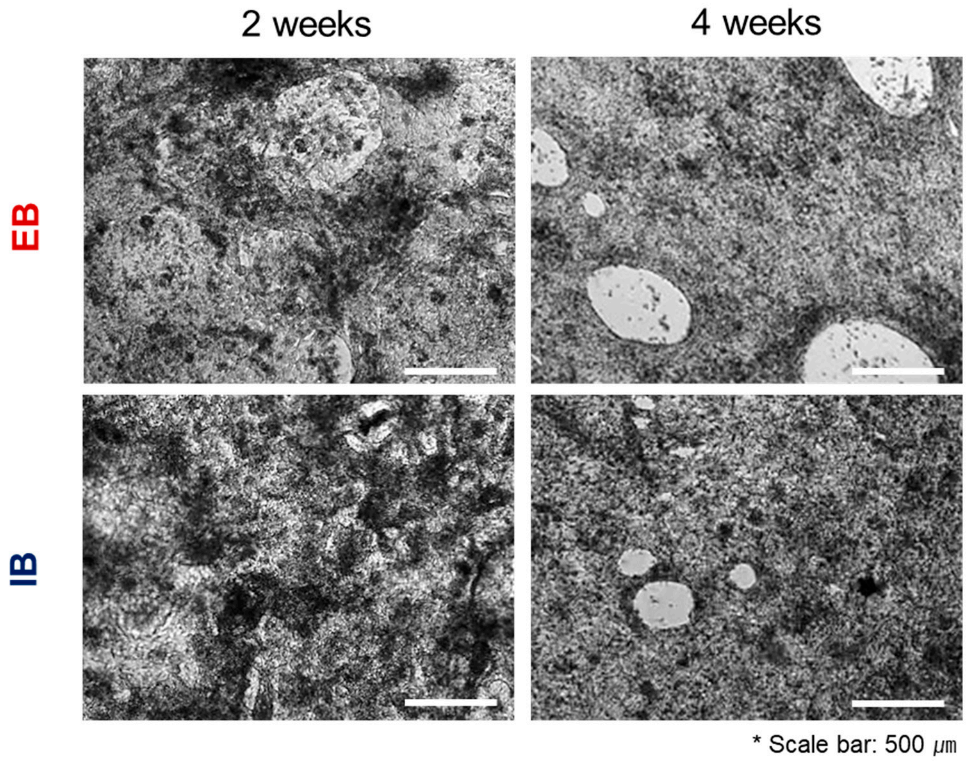

Video S1. Beating video of hiPSC-CM.
